# Supplementary material for: The pore structure of Clostridium perfringens epsilon toxin
Source: Nat Commun. 2019 Jun 14;10:2641. doi: 10.1038/s41467-019-10645-8 (PMC6572795; doi:10.1038/s41467-019-10645-8)
Supplement: Supplementary file 1 — Supplementary Information [file 41467_2019_10645_MOESM1_ESM.pdf]

The pore structure of *Clostridium perfringens* epsilon toxin

Savva *et al.*

Supplementary Information

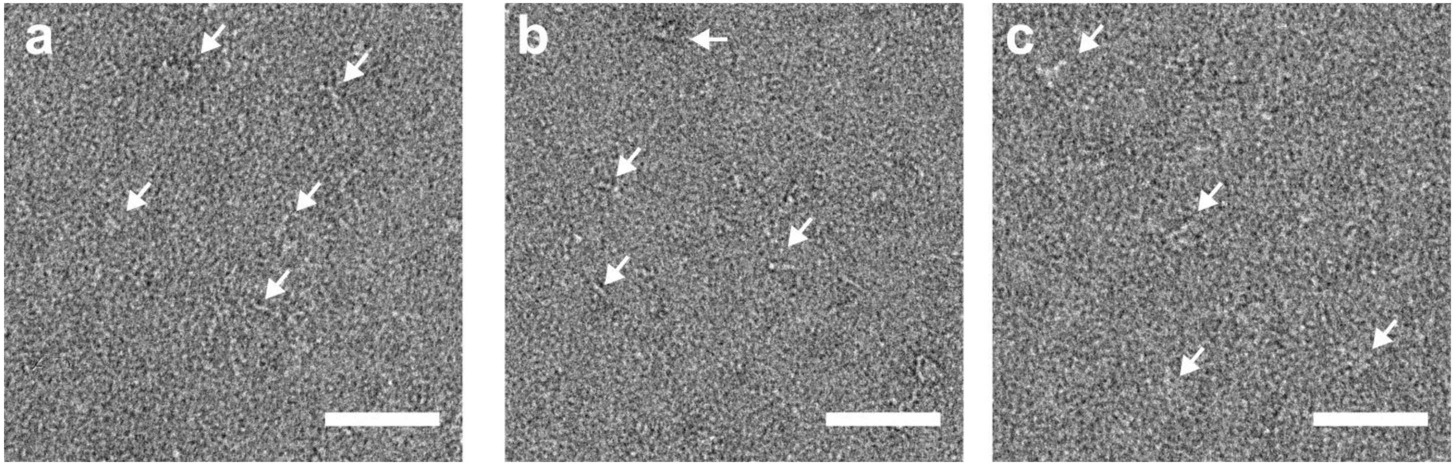

Supplementary Figure 1. Negative stain electron microscopy of Etx variants. Trypsin activated Etx variants were negatively stained and observed by electron microscopy. (**a**) Etx wild type, (**b**) Etx-H162A and (**c**) Etx-D250A. No regular oligomeric species were observed apart from possible monomers (~30 kDa) and larger protein clumps (marked by arrows). Scale bars correspond to 50 nm.

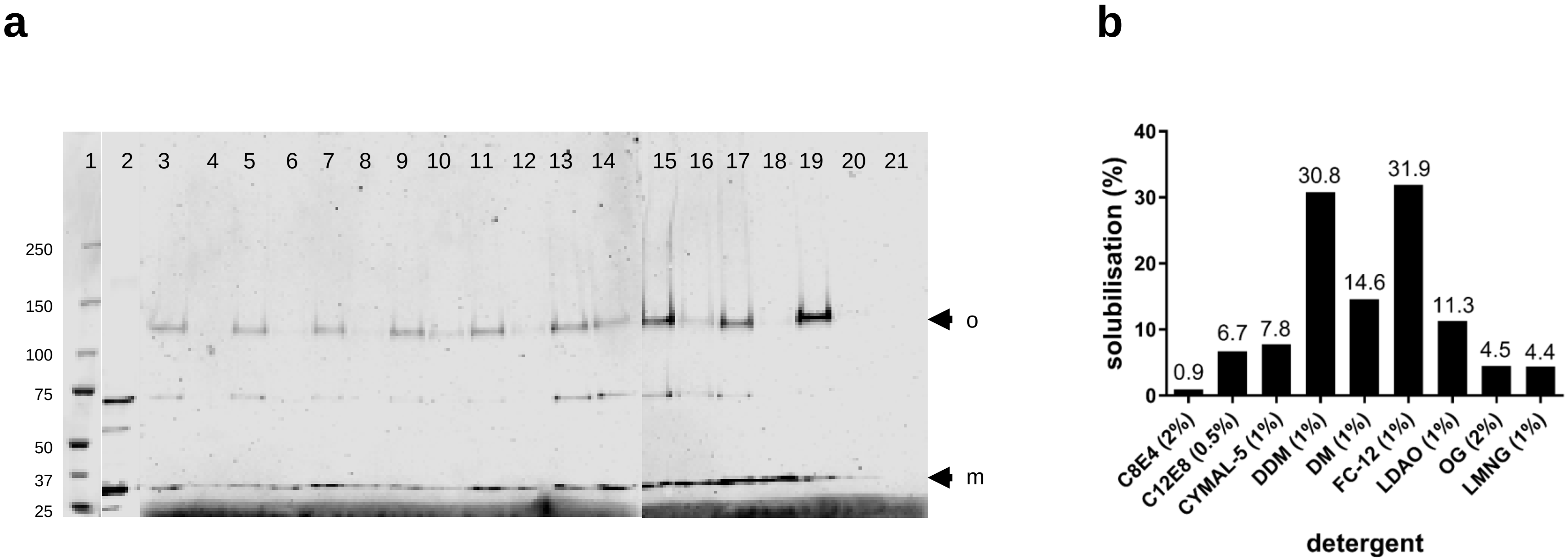

Supplementary Figure 2. Detergent extraction and solubilisation of Etx pores. (a) Super Dome cell membrane was incubated with trypsin-activated recombinant Etx and membrane incubations were distributed between nine tubes for detergent screening. After ultracentrifugation, each pellet was resuspended in detergent solution and incubated overnight at 4 °C. Following detergent extraction, samples containing total protein in detergent (total) were removed from each tube for Western blot analysis. After ultracentrifugation, insoluble material was removed and the supernatants were collected to assess Etx pore solubilisation by Western blot analysis. Lane 1: Molecular weight marker, Lane 2: Trypsin-activated recombinant Etx, Lanes 3-20: Etx plus Super Dome membrane, Lanes 3-4: C8E4 total and supernatant, Lanes 5-6: C12E8 total and supernatant, Lanes 7-8: CYMAL-5 total and supernatant, Lanes 9-10: DDM total and supernatant, Lanes 11-12: DM total and supernatant, Lanes 13-14: FC-12 total and supernatant, Lanes 15-16: LDAO total and supernatant, Lanes 17-18: OG total and supernatant, Lanes 19-20: LMNG total and supernatant. Lane 21: Super Dome membrane. Arrows indicate Etx monomer (m) and Etx oligomer (o) The molecular masses (kDa) of protein standards are shown to the left of the blot. **(b)** The solubilization yields for each detergent were determined by calculating the % of Etx pore in the supernatant relative to its amount in the total protein sample using Image Studio ver 5.2 software. Source data are provided as a Source Data file.

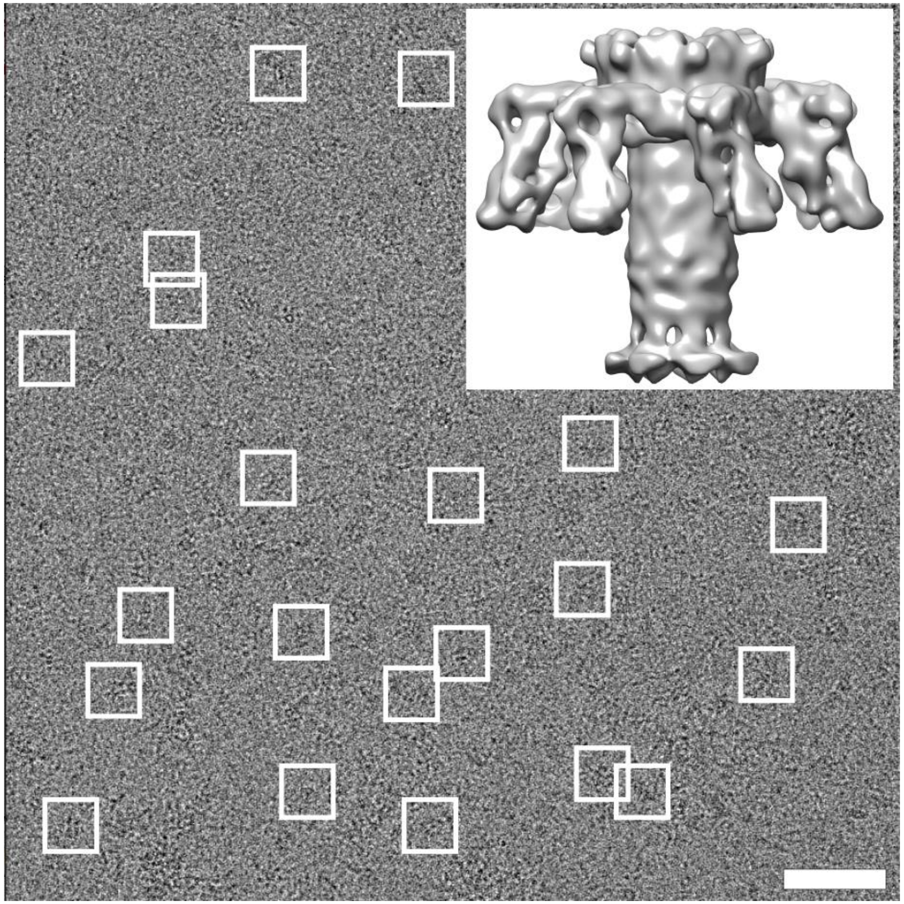

Supplementary Figure 3. Conventionally defocused (Δz = -1.8 µm) electron micrograph of Etx pores. Inset indicates the 3D reconstruction to 4.6 Å from ~14,000 particles. Example particles that contributed to the final map are highlighted. Scale bar corresponds to 50 nm.

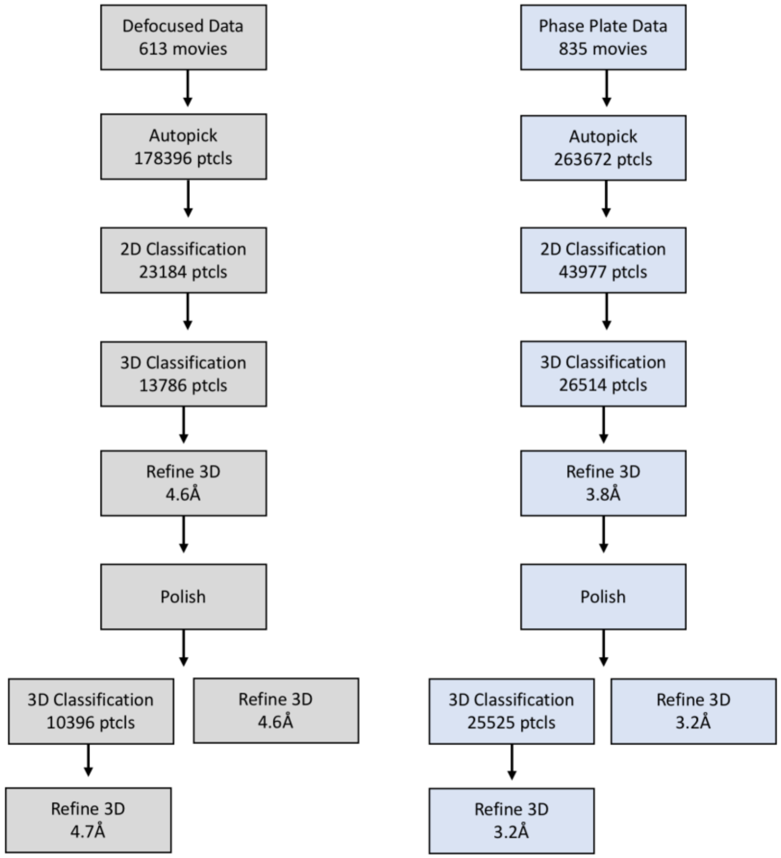

Supplementary Figure 4. Summary of the image processing procedures followed in Relion 3 for the conventionally defocused and phase plate collected datasets.

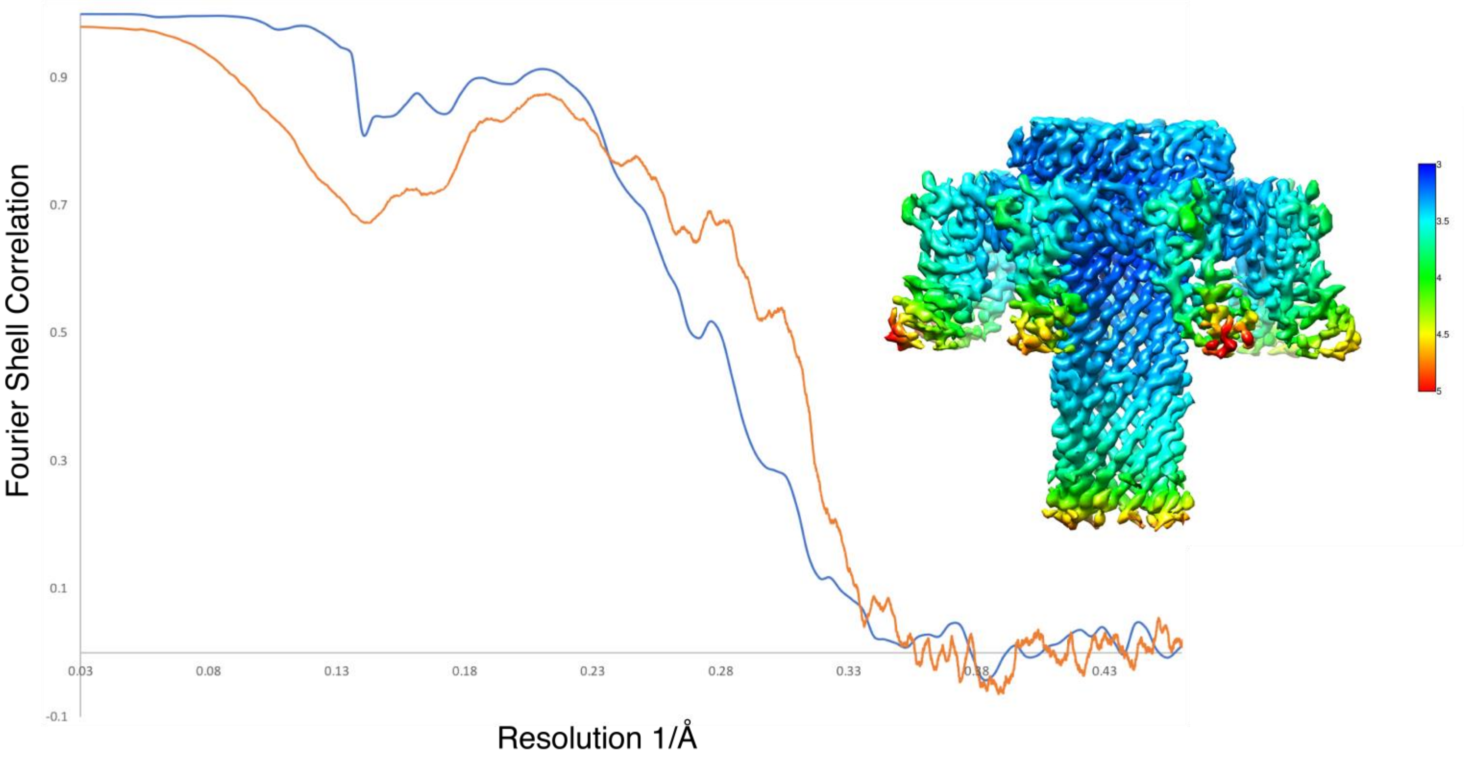

Supplementary Figure 5. Resolution assessment of the Etx map. Fourier Shell Correlation between the two half-maps (blue) and between the sharpened map and the refined model (orange). Local resolution calculated from the unsharpened, unfiltered map. The resolution is lower near the tips of the receptor binding domains and the β-turn of the β-barrel near the intracellular side.

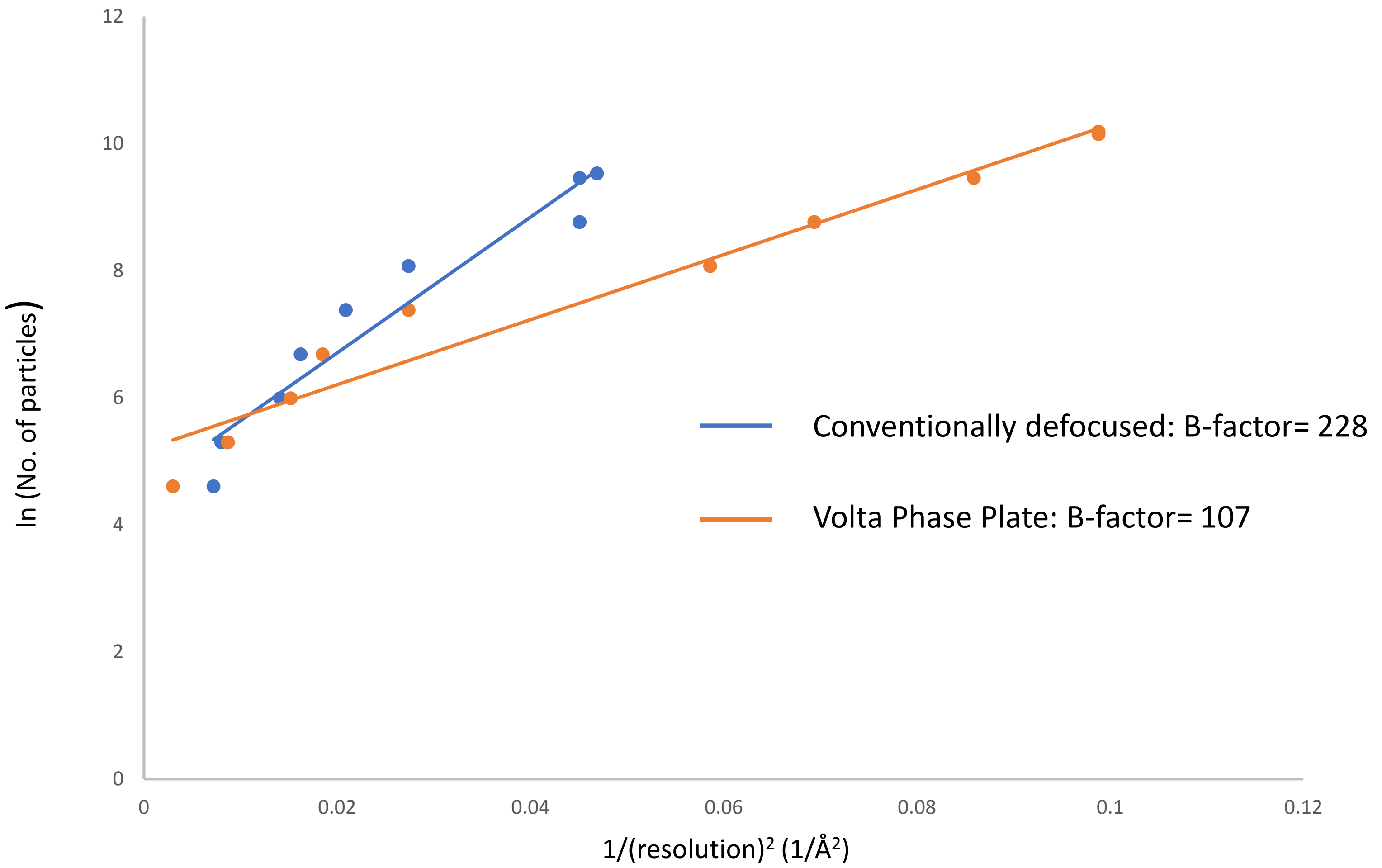

Supplementary Figure 6. Data quality assessment of the conventionally defocused and phase plate collected datasets. The exact same grid was used to collect the data in similar areas. The phase plate data was collected second.

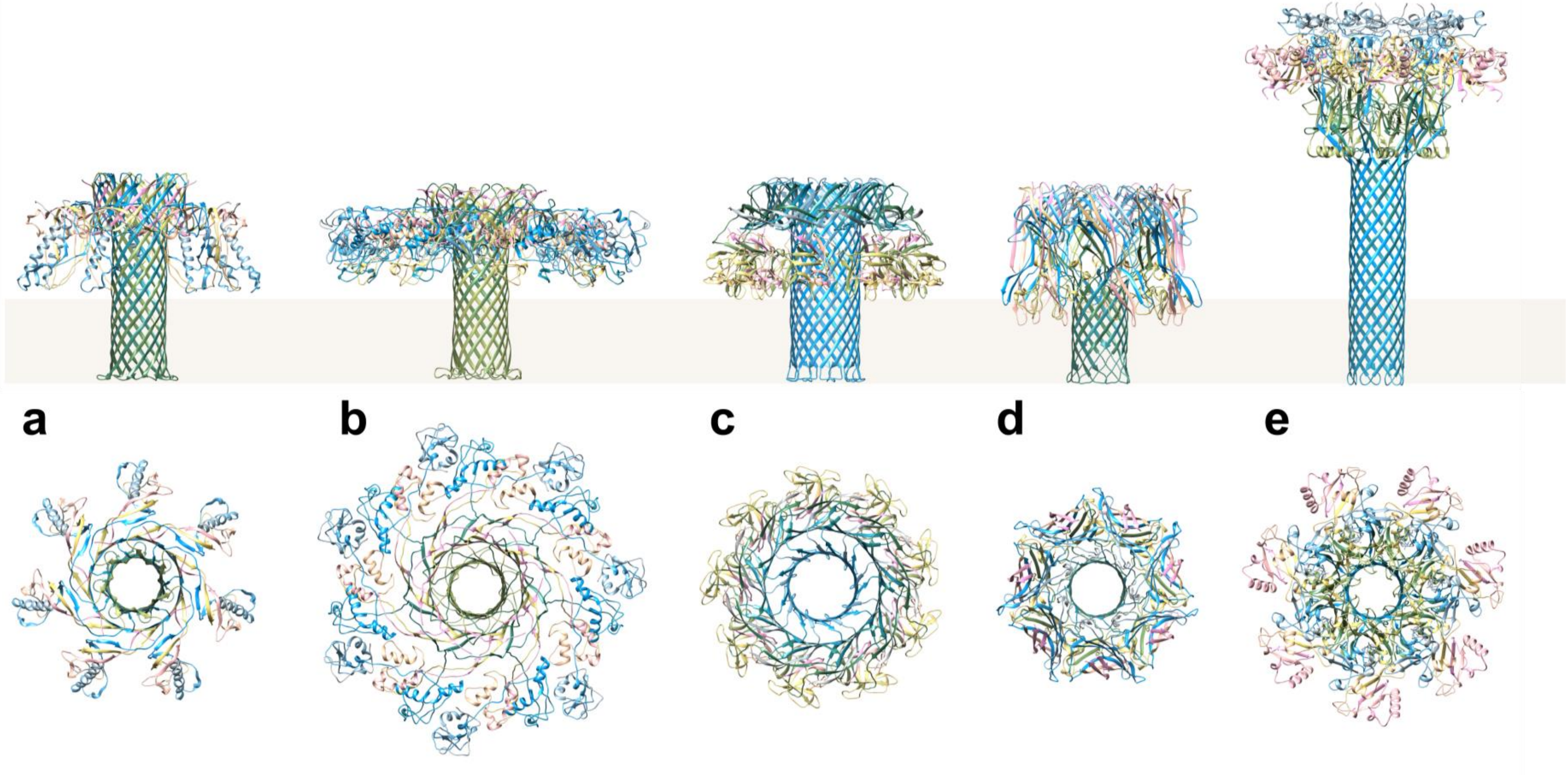

Supplementary Figure 7. Comparison of the aerolysin family members and other small β-PFT families. Epsilon toxin (**a**, 6RB9), Aerolysin (**b**, 5JZT), Lysenin (**c**, 5GAQ), α-hemolysin (**d**, 7AHL) and Anthrax protective antigen (**e**, 3J9C). The approximate borders of the lipid bilayer are shown in light brown.

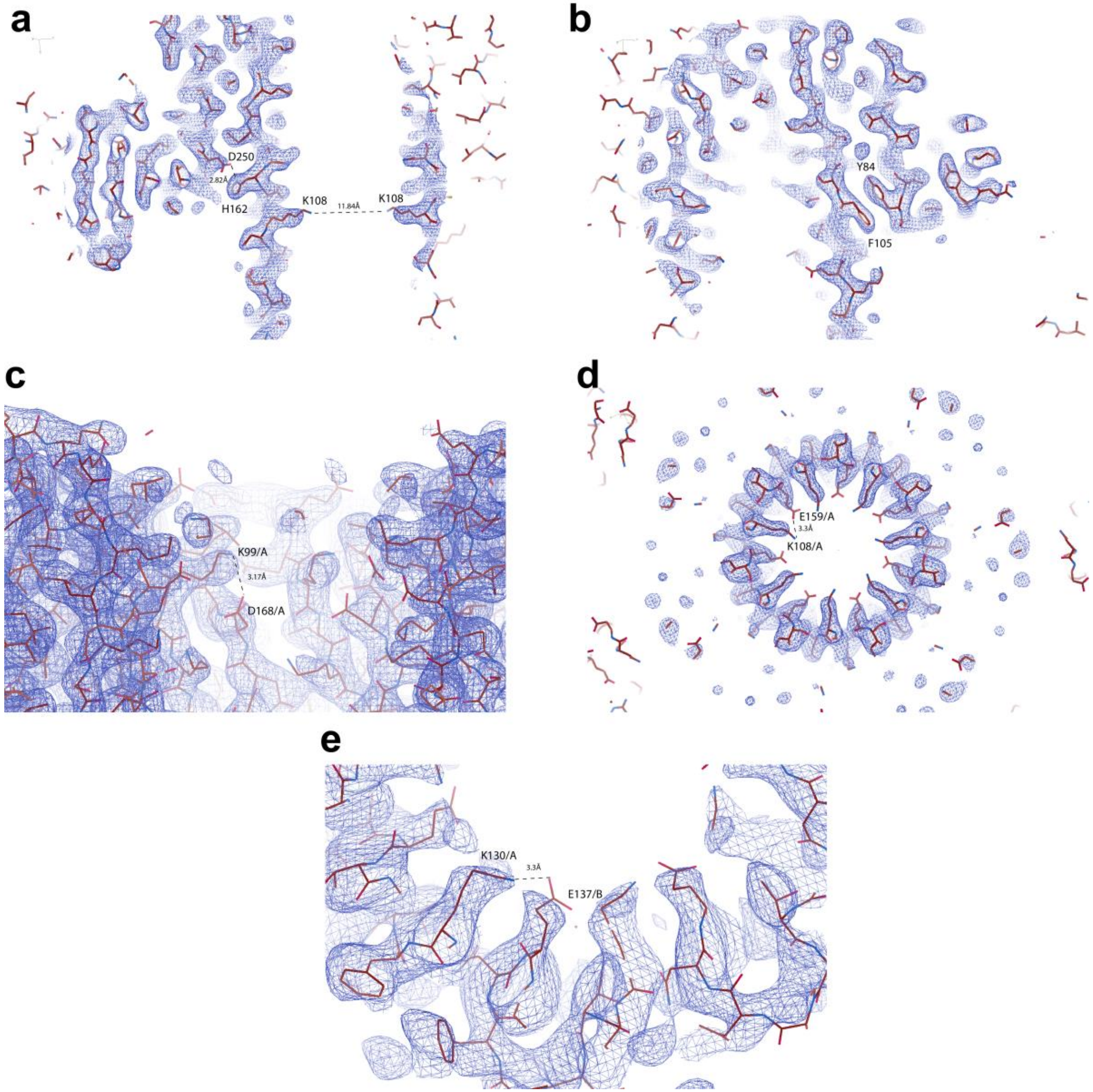

Supplementary Figure 8. Cryo-EM map density and atomic model of important residues. (**a**) The hydrogen bond between H162 in the inner β-barrel and D250 in the cap domain. The narrowing of the pore lumen by K108 is also shown. (**b**) π-stacking between F105 in the inner β-barrel and Y84 in the cap domain, as well as other interactions along the two domains are shown. Interactions between the cap domain outer-β-barrel and the inner β-barrel may help in stabilising the pore conformation. Intra-chain (**c-d**) and inter-chain (**e**) salt bridges formed in the inner β-barrel.

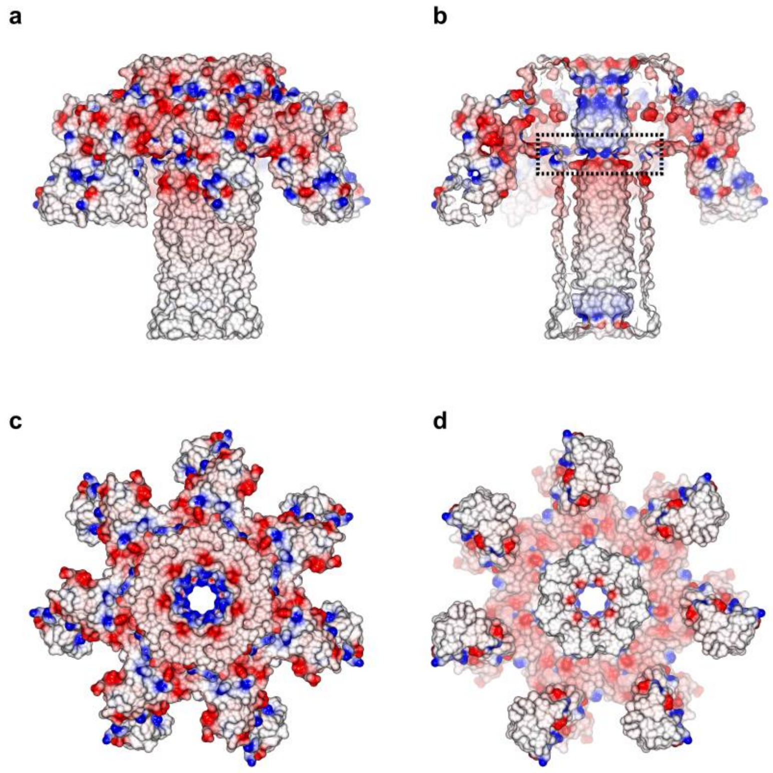

Supplementary Figure 9. Electrostatic surface potential of the Etx pore. The surface charge distribution is highlighted from the side ((**a**), outer surface and (**b**), internal lumen). The lumen restriction by K108 is highlighted by the rectangle in (**b**). Views from the extracellular (**c**) and intracellular (**d**) sides are indicated. Colour code: red: negative charge; blue: positive charge: grey: uncharged

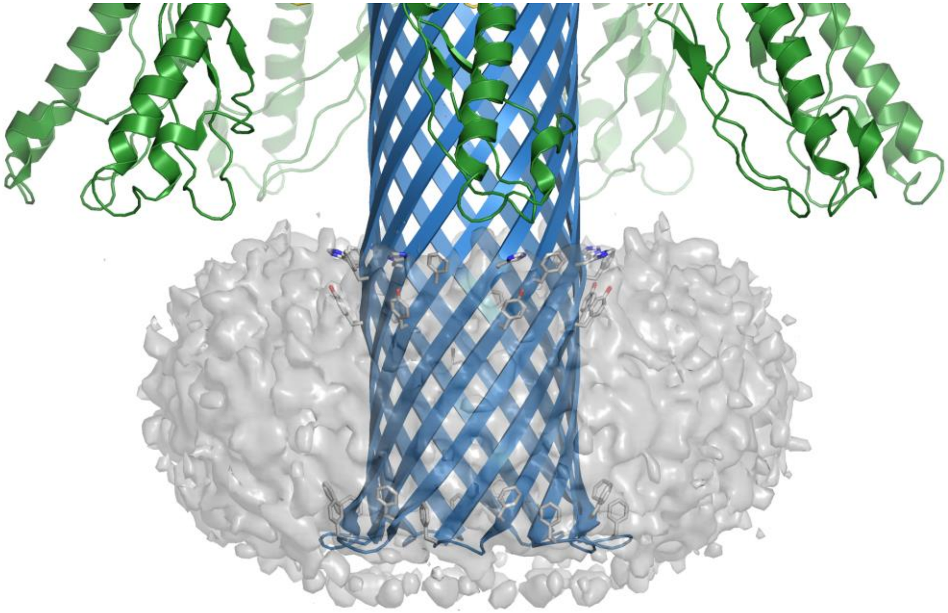

Supplementary Figure 10. Cryo-EM density for the detergent micelle. The aromatic belts on the central β-barrel (highlighted in Fig. 3c and shown here in stick representation) are immersed inside the detergent micelle, suggesting these residues will be membrane embedded. Colouring scheme as in Fig. 3. Detergent micelle shown in transparent grey.

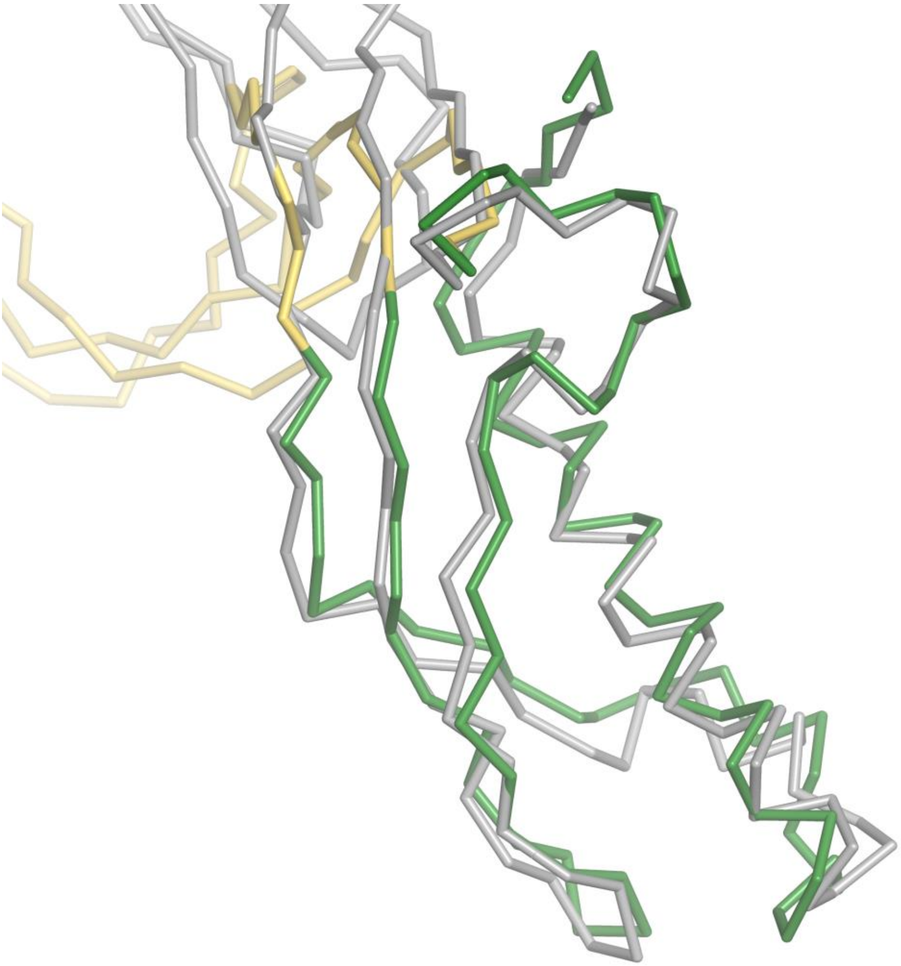

Supplementary Figure 11. Superposition of the RBD of the water-soluble monomer and pore form of Etx. The RBD of the water-soluble monomer (grey) undergoes minimal changes in the transition to the membrane-inserted form (green) with an RMSD of 1.7 Å.

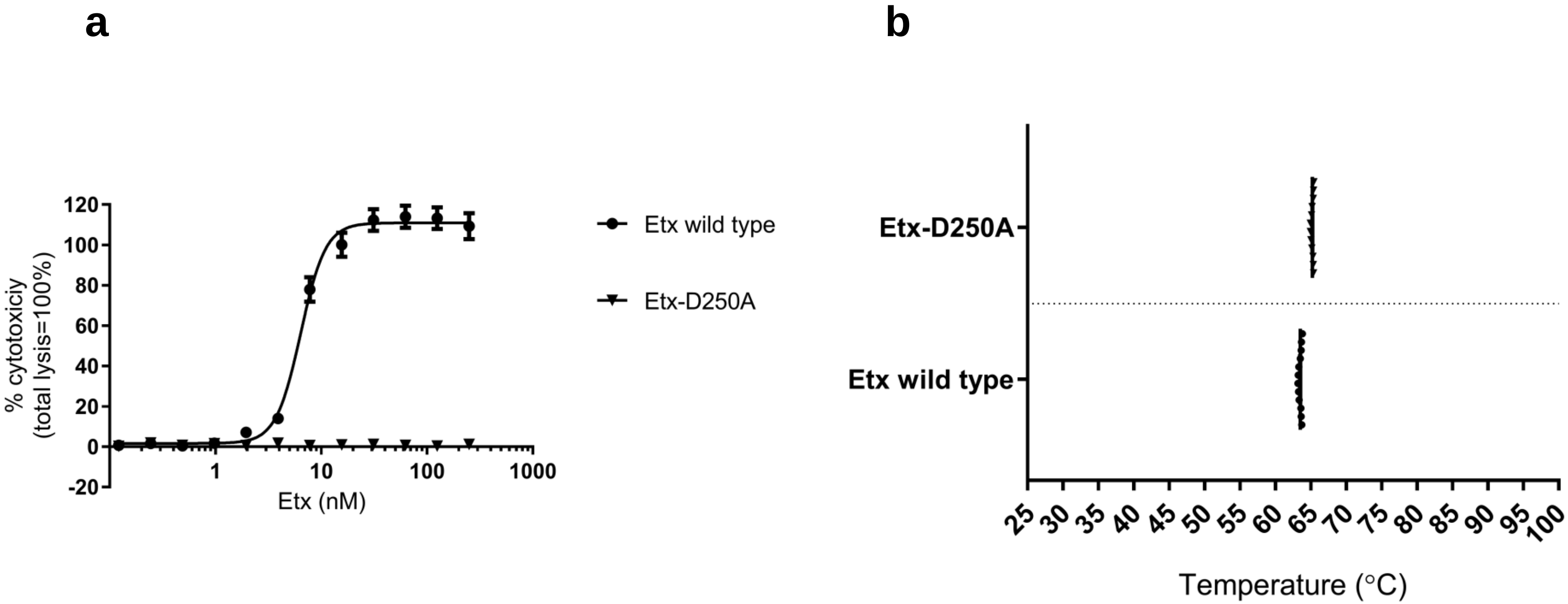

Supplementary Figure 12. Recombinant Etx-D250A is inactive. (**a**) The cytotoxic activity of trypsin-activated Etx-D250A or Etx wild type towards Super Dome cells was determined by measuring the release of lactate dehydrogenase (LDH) from lysed cells. Results were normalised to the signal from cells treated with PBS only (0% lysis) and cells treated with 0.9% (v/v) Triton X-100 (100% lysis). Results are presented as the mean of triplicate assays, each performed in duplicate (± S.E.M.). Source data are provided as a Source Data file. (**b**) Thermo-stability of Etx-D250A and Etx wild type was determined by the Boltzmann method using the Protein Thermal Shift software (Applied Biosystems). Scatter dot plot represents the mean of duplicate assays each performed in six replicates (± S.E.M.). Source data are provided as a Source Data file.

a

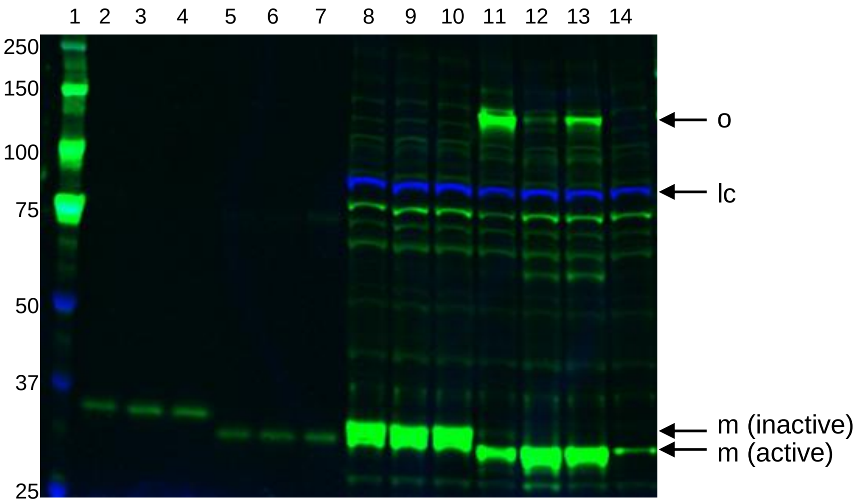

b

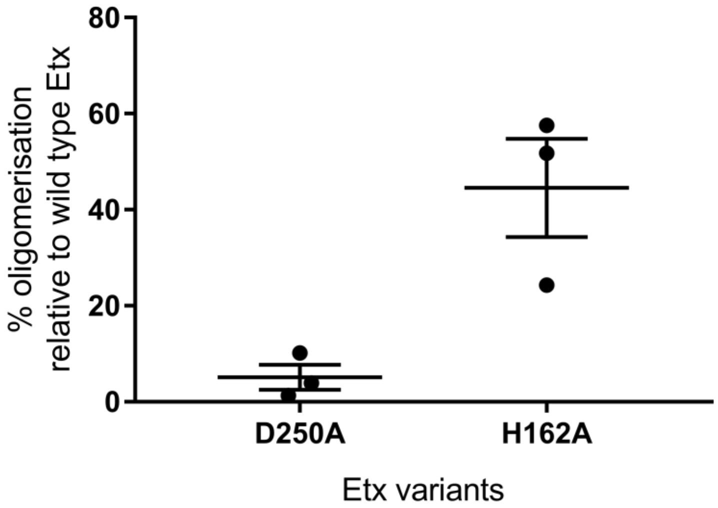

Supplementary Figure 13. SDS-PAGE analysis of oligomer formation. (**a**) Super Dome cells were incubated with P-Etx, trypsin-activated Etx or buffer only at 37 °C for 1 h. Following detergent solubilisation (1% (w/v) DDM), toxin complexes (30 µg per lane) or monomers were separated by SDS-PAGE and oligomerization was assessed by immunoblotting with anti-Etx polyclonal antibody. Na+/K+-ATPase α1 (C464.6): sc-21712 antibody was used as loading control. Lane 1: Molecular weight marker; Lanes 2-4: P-Etx wild type, P-Etx-D250A and P-Etx-H162A, respectively; Lanes 5-7: trypsin-activated Etx wild type, Etx-D250A and Etx-H162A, respectively; Lanes 8-10: Super Dome cells incubated with P-Etx wild type, P-Etx-D250A or P-Etx-H162A, respectively; Lanes 11-13: Super Dome cells incubated with trypsin-activated Etx wild type, Etx-D250A or Etx-H162A, respectively; Lane 14: Super Dome cells incubated with buffer only. Arrows indicate Etx monomer (m), Etx oligomer (o) and loading control (lc). One representative immunoblot out of three is shown. The molecular masses (kDa) of protein standards are shown to the left of the blot. **(b)** Normalised, mean oligomer yields for Etx-D250A and Etx-H162A were determined by calculating the % oligomerisation relative to cells treated with wild type Etx (100%) using Image Studio ver 5.2 software. Values represent means (± S.E.M.) from triplicate experiments. Source data are provided as a Source Data file.

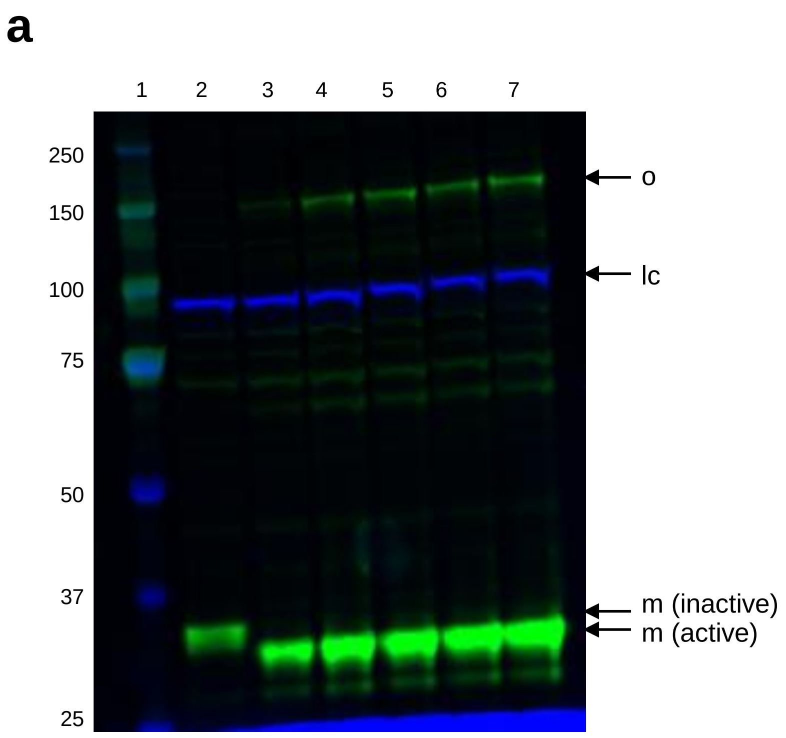

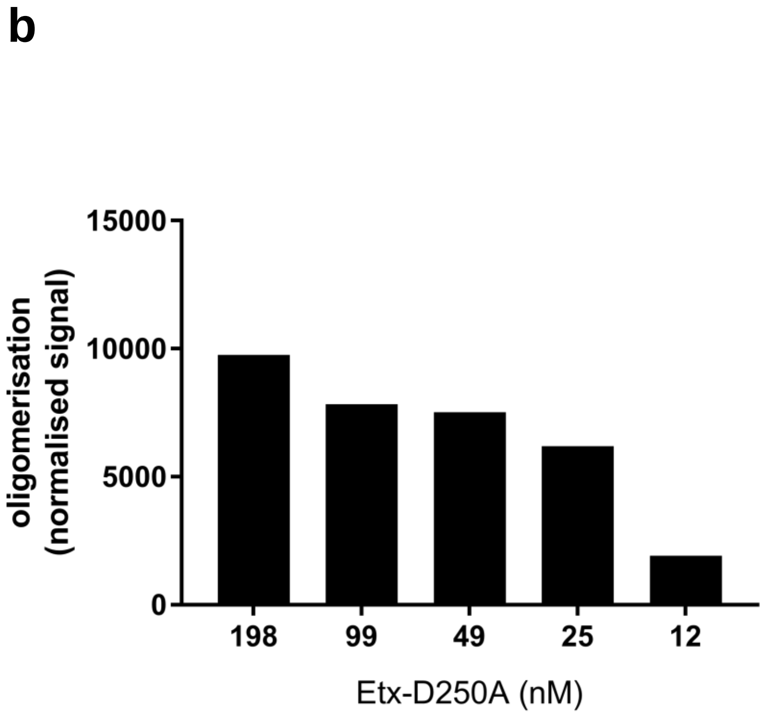

Supplementary Figure 14. Etx-D250A forms oligomers in a concentration-dependent manner. (**a**) Super Dome cells were incubated with P-Etx-D250A or a two-fold dilution series of trypsin-activated Etx-D250A (ranging from 198 nM to 12 nM) at 37 °C for 1 h. Following detergent solubilisation, toxin complexes were separated by SDS-PAGE (30 µg per lane) and oligomerization was assessed by immunoblotting with anti-Etx polyclonal antibody. Na+/K+-ATPase α1 (C464.6): sc-21712 antibody was used as loading control. Lane 1: Molecular weight marker; Lane 2: Super Dome cells incubated with P-Etx-D250A; Lanes 3-7: Super Dome cells incubated with increasing concentrations of trypsin-activated Etx-D250A. Arrows indicate Etx monomer (m), Etx oligomer (o) and loading control (lc). The molecular masses (kDa) of protein standards are shown to the left of the blot. **(b)** The oligomerisation yield for Etx-D250A at each dose was determined using Image Studio ver 5.2 software. Source data are provided as a Source Data file.

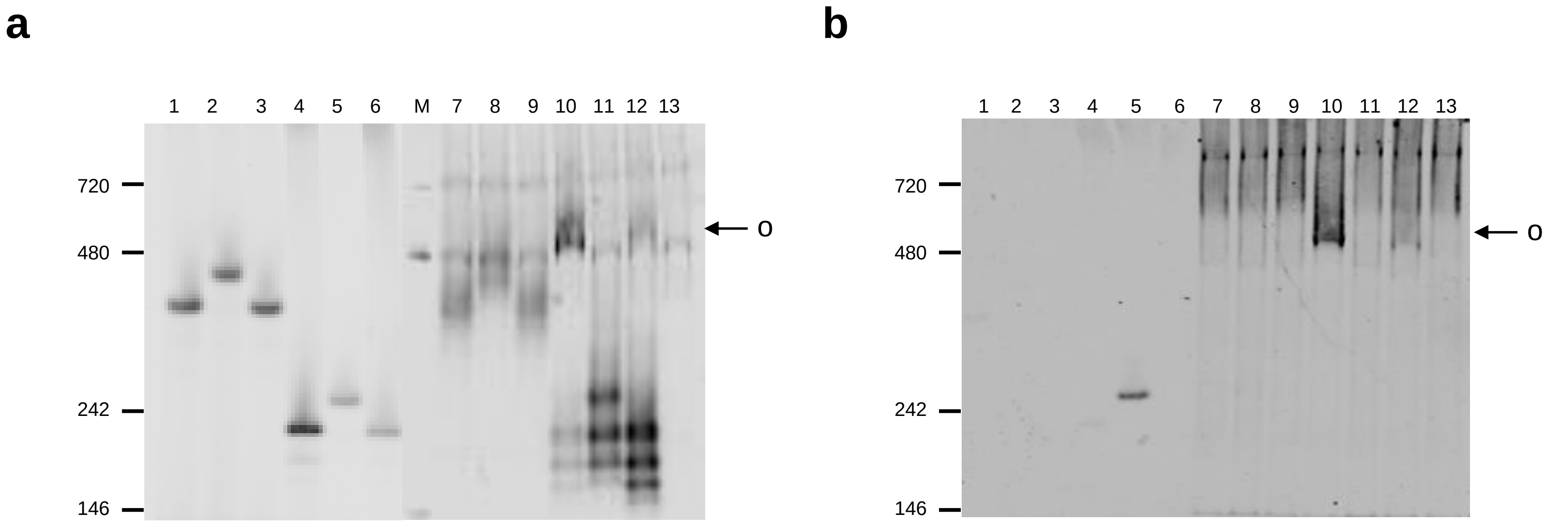

Supplementary Figure 15. Native-PAGE analysis of oligomer formation. Super Dome cells were incubated with P-Etx, trypsin-activated Etx or buffer only at 37 °C for 1 h. Following detergent solubilisation (1% (w/v) DDM), toxin complexes (30 µg per lane) or monomers were separated using native-PAGE and oligomerization was assessed by immunoblotting with anti-Etx polyclonal antibody (**a**) or anti-His monoclonal antibody (**b**). Lanes 1-3: P-Etx wild type, P-Etx-D250A or P-Etx-H162A, respectively; Lanes 4-6: trypsin-activated Etx wild type, Etx-D250A or Etx-H162A, respectively; Lanes 7-9: Super Dome cells incubated with P-Etx wild type, P-Etx-D250A or P-Etx-H162A, respectively; Lanes 10-12: Super Dome cells incubated with trypsin-activated Etx wild type, Etx-D250A or Etx-H162A, respectively; Lane 13: Super Dome cells incubated with buffer only. Lane M: Molecular weight marker. Arrows indicate Etx oligomer (o). One representative immunoblot out of three is shown. The molecular masses (kDa) of protein standards are shown to the left of each blot. Source data are provided as a Source Data file.

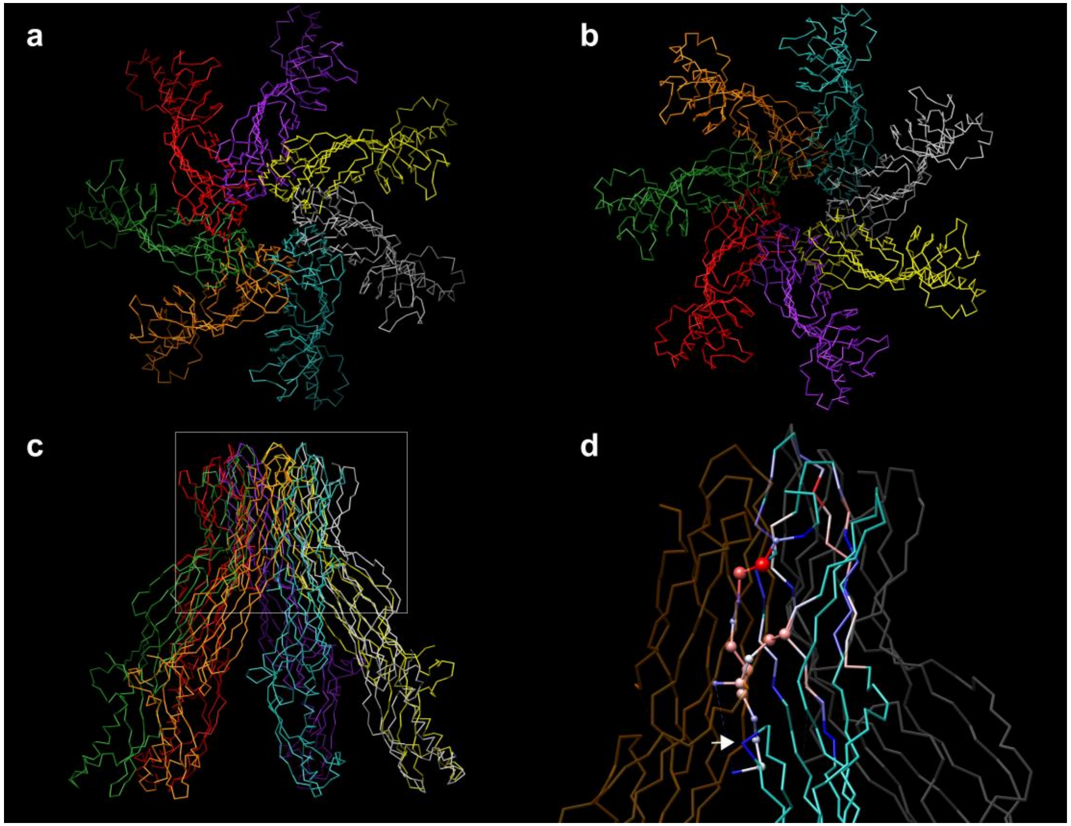

Supplementary Figure 16. Hypothetical Etx pre-pore. The pre-pore was assembled by imposing 7-fold symmetry to the Etx crystal structure which was aligned to the receptor binding domain of each monomer in the pore structure. Views from the extracellular (**a**) and cytoplasmic views (**b**). Side view (**c**) and closeup with only 3 chains shown for clarity (**d**). The molecular clashes are shown coloured blue to red from less to more severe. The CTP is highlighted in ball and stick representation with larger balls indicating more severe clashes. The majority of these clashes are derived from the CTP, which has to be removed prior to oligomerisation, and in the interactions with the neighbouring monomers, which indicates a rearrangement must occur upon pre-pore formation. The white arrow points to the insertion loop or “tongue” that will also have to be dislocated in the pre-pore.

Primer	*Primer sequence*[a]
Etx-*Nco*I-F	catg*ccatgg*cttcttatgataatgtagatac
Etx-*Xho*I-R	ccg*ctcgag*ttattttattcctggtgccttaatat
D250A-F	gaaaaggaaattatagtgcagttatggga**gcg**gagttaatagttaaggttagaaatttaaat
D250A-R	atttaaatttctaaccttaactattaactc**cgc**tcccataactgcactataatttccttttc
H162A-F	caaatacaaatacaaatactaattcaaaagaaattact**gcg**aatgtcccttcacaagatatacta
H162A-R	tagtatatcttgtgaagggacatt**cgc**agtaatttcttttgaattagtatttgtatttgtatttg

Supplementary Table 1. Synthetic oligonucleotides used in this study.
[a]Nucleobases in italics are restriction enzyme sites and nucleobases in bold are the codons used for substitution. All primer sequences are shown in 5’ to 3’ orientation.
