# Supplementary material for: The pore structure of Clostridium perfringens epsilon toxin
Source: Nat Commun. 2019 Jun 14;10:2641. doi: 10.1038/s41467-019-10645-8 (PMC6572795; doi:10.1038/s41467-019-10645-8)
Supplement: Supplementary file 3 — Reporting Summary [file 41467_2019_10645_MOESM3_ESM.pdf]

nature research

Corresponding author(s): *Double-blind peer review submissions: write DBPR and your manuscript number here instead of author names.*

Last updated by author(s): *YYYY-MM-DD*

Reporting Summary

Nature Research wishes to improve the reproducibility of the work that we publish. This form provides structure for consistency and transparency in reporting. For further information on Nature Research policies, see Authors & Referees and the Editorial Policy Checklist.

Statistics

For all statistical analyses, confirm that the following items are present in the figure legend, table legend, main text, or Methods section.

n/a	Confirmed	
☐	☒	The exact sample size (*n*) for each experimental group/condition, given as a discrete number and unit of measurement
☐	☒	A statement on whether measurements were taken from distinct samples or whether the same sample was measured repeatedly
☐	☒	The statistical test(s) used AND whether they are one- or two-sided<br>*Only common tests should be described solely by name; describe more complex techniques in the Methods section.*
☐	☒	A description of all covariates tested
☐	☒	A description of any assumptions or corrections, such as tests of normality and adjustment for multiple comparisons
☐	☒	A full description of the statistical parameters including central tendency (e.g. means) or other basic estimates (e.g. regression coefficient) AND variation (e.g. standard deviation) or associated estimates of uncertainty (e.g. confidence intervals)
☐	☒	For null hypothesis testing, the test statistic (e.g. *F*, *t*, *r*) with confidence intervals, effect sizes, degrees of freedom and *P* value noted<br>*Give P values as exact values whenever suitable.*
☐	☒	For Bayesian analysis, information on the choice of priors and Markov chain Monte Carlo settings
☐	☒	For hierarchical and complex designs, identification of the appropriate level for tests and full reporting of outcomes
☐	☒	Estimates of effect sizes (e.g. Cohen's *d*, Pearson's *r*), indicating how they were calculated

Our web collection on statistics for biologists contains articles on many of the points above.

Software and code

Policy information about availability of computer code

Data collection

Data was collected on a FEI Titan Krios operating at an accelerating voltage of 300 kV. All datasets were collected on a FEI Falcon 3EC direct electron detector at a nominal magnification of 75Kx (calibrated pixel size of 1.07 Å) and a dose rate of 0.6e- per pixel per second in counting mode over 60 seconds and 75 fractions.

Data analysis

Conventionally defocused and phase plate datasets were collected using the EPU software (Thermo-Fisher Scientific).

All data datasets were processed in Relion 3. Micrographs were first corrected for large movements using MotionCorr2 and a 4 x 4 patch with no grouping. CTF parameters were estimated using GTCF. Autopicking was performed in Relion after creating references from manually picked particles. The processing procedure is summarized in Supplementary Figure 4. The resolution was calculated using Gold-Standard 0.143 criterion, resulting in a 4.6 Å map for the defocused dataset and 3.2 Å for the phase plate dataset. The variation in resolution in the map was calculated using the Relion local resolution implementation. Box sizes of 220 pixels were used for both the defocused and phase plate datasets. To ensure that high resolution information was not lost by the high defocus values used for the former dataset, particles were re-extracted in 400 pixel boxes and subjected to 3D refinement. No increase in resolution was observed for the larger box size particles. B-factor plots were calculated using the bfactor_plot.py scripts provided with Relion 3.

A model was built into the 3.2 Å map by initially docking the receptor-binding domain of wild type Etx crystal structure (PDB: 1UYJ) and then extending this using Coot. Refinement of the atomic coordinates was performed using Phenix real space refine and re-iterating the model building-refinement procedure several times.

Thermostability of purified recombinant epsilon toxin mutant D250A was analysed using the Protein Thermal Shift Software (Applied Biosystems, USA) to calculate the melting temperature (Tm) using the Boltzmann method.

The toxin dose required to kill 50% of the cell monolayer (CT50) was determined by nonlinear regression analysis, fitting a variable slope log(dose) versus response curve, constraining F to a value of 50 (logCT50=logCTF - (1/HillSlope)*log(F/(100-F)). Cytotoxicity data were analysed using Prism v7 software (GraphPad Software, Inc., La Jolla, CA, USA).

Epsilon pore solubilization yields for each detergent were determined by calculating the % of epsilon pore in the supernatant relative to its amount in the total protein sample using Image Studio ver 5.2 software (LI-COR Biosciences, Lincoln, USA).

Oligomerisation yields were determined using the Image Studio ver 5.2 software (LI-COR Biosciences, Lincoln, USA) and the Housekeeping Protein Normalization Protocol (LI-COR Biosciences; https://www.licor.com/documents/kijrkoh8oqwv212577slsljldl74og16). The oligomer yields for each Etx variant were determined by calculating the percentage of fluorescence intensity relative to wild type toxin.

For manuscripts utilizing custom algorithms or software that are central to the research but not yet described in published literature, software must be made available to editors/reviewers. We strongly encourage code deposition in a community repository (e.g. GitHub). See the Nature Research guidelines for submitting code & software for further information.

Data

Policy information about availability of data

All manuscripts must include a data availability statement. This statement should provide the following information, where applicable:

- Accession codes, unique identifiers, or web links for publicly available datasets
- A list of figures that have associated raw data
- A description of any restrictions on data availability

Data supporting the findings of this manuscript are available from the corresponding author upon reasonable request. A reporting summary for this Article is available as a Supplementary Information file.
The cryo-EM maps of epsilon toxin pore have been deposited to the Electron Microscopy Data Bank under accession number EMD-4789 and the refined atomic coordinates have been deposited to the Protein Data Bank under accession number 6RB9. The source data for Figure 1 and Supplementary Figures 2, 12-15 are provided as a Source Data file.

Field-specific reporting

Please select the one below that is the best fit for your research. If you are not sure, read the appropriate sections before making your selection.

☒ Life sciences ☐ Behavioural & social sciences ☐ Ecological, evolutionary & environmental sciences

For a reference copy of the document with all sections, see nature.com/documents/nr-reporting-summary-flat.pdf

Life sciences study design

All studies must disclose on these points even when the disclosure is negative.

Sample size	No sample-size calculation was performed.<br><br>An initial dataset of 613 micrographs resulted in approximately 14,000 particles after 2D and 3D classification clean-up steps and produced a map which reached 4.6 Å, at which resolution the β-strands just begin to separate. We reasoned that the use of a Volta Phase Plate (VPP) would be advantageous for two reasons. First, the contrast of the protein component would increase, aiding particle picking and alignment. Second, the number of particles required to achieve a high-resolution structure could decrease as compared to conventional defocused data. Therefore, we proceeded to image the exact same grid used before with the VPP. Although the samples still appeared heterogeneous, approximately two times as many particles remained after clean-up steps as compared to the non-VPP dataset from a dataset that was only 25% larger. This resulted in a final map at an overall resolution of 3.2 Å. Estimation of the B-factors for both datasets indicated that the phase plate data was indeed of higher quality when comparing the same number of particles used in a reconstruction.
Data exclusions	No data were excluded from the analyses.
Replication	Cytotoxicity measurements for recombinant wild type epsilon toxin were performed in triplicate and each assay was performed in triplicate. Cytotoxicity assays for recombinant Etx-D250A mutant was performed in triplicate and each assay was performed in duplicate. Thermostability measurements are presented as the the mean of duplicate assays each performed in six replicates. Oligomer stability assays are presented as the the mean of triplicate experiments. All attempts at replication were successful.
Randomization	The cytotoxic activity of recombinant wild type epsilon toxin towards Super Dome cells was determined by measuring the release of lactate dehydrogenase (LDH) from lysed cells. Results were normalised to the signal from cells treated with PBS only (0% lysis) and cells treated with 0.9% (v/v) Triton X-100 (100% lysis).
Blinding	Blinding was not relevant to our study. The image processing procedure is summarized in Supplementary Figure 4.

Reporting for specific materials, systems and methods

We require information from authors about some types of materials, experimental systems and methods used in many studies. Here, indicate whether each material, system or method listed is relevant to your study. If you are not sure if a list item applies to your research, read the appropriate section before selecting a response.

Materials & experimental systems

n/a	Involved in the study
☐	☒ Antibodies
☐	☒ Eukaryotic cell lines
☒	☐ Palaeontology
☒	☐ Animals and other organisms
☒	☐ Human research participants
☒	☐ Clinical data

Methods

n/a	Involved in the study
☒	☐ ChIP-seq
☒	☐ Flow cytometry
☒	☐ MRI-based neuroimaging

Antibodies

Antibodies used	To assess Etx pore solubilisation by Western blot analysis after detergent screening, we used 6x-His Tag Monoclonal Antibody (4A12E4) (Invitrogen; catalog number 37-2900; Lot number RL231619) and IRDye® 800CW Goat (polyclonal) Anti-Mouse IgG (H+L), Highly Cross Adsorbed secondary antibody (LI-COR Biosciences; catalog number 926-32210, Lot # C60726-02) at 1:500 and 1:5,000 dilutions, respectively.<br>To assess stability of oligomeric complexes by Western blot, we used the polyclonal antibody against epsilon toxin mutant Y30A-Y196A raised in rabbits as described in: Bokori-Brown M, et al. Clostridium perfringens epsilon toxin mutant Y30A-Y196A as a recombinant vaccine candidate against enterotoxemia. Vaccine 32, 2682-2687 (2014), and IRDye® 800CW goat (polyclonal) anti-rabbit IgG (H+L) secondary antibody (LI-COR Biosciences; catalog number 926-32211, Lot number C80718-15) at 1:500 and 1:5,000 dilutions, respectively. For loading control we used the mouse monoclonal Na+/K+ - ATPase α1 (C464.6): sc-21712 primary antibody (Santa Cruz Biotechnology; catalog number sc-21712; Lot number F2018) and IRDye® 680RD goat (polyclonal) anti-mouse IgG (H+L) secondary antibody (LI-COR Biosciences; catalog number 925-68070; Lot number C80926-19) at 1:200 and 1:5,000 dilution, respectively.
Validation	Histidine tagged (6xHis) epsilon pore was detected by Western blot using 6x-His Tag Monoclonal Antibody (4A12E4) at 1:500 dilution according to the manufacturer's recommendation (https://www.thermofisher.com/order/genome-database/generatePdf?productName=6x-His Tag&assayType=PRANT&detailed=true&productId=37-2900) followed by IRDye® 800CW Goat (polyclonal) Anti-Mouse IgG (H+L) Highly Cross Adsorbed secondary antibody at 1:5000 dilution according to the manufacturer's recommendations (LI-COR Biosciences). The conjugate has been specifically tested and qualified for Western blot.<br>To assess stability of oligomeric complexes by Western blot we used the polyclonal antibody against epsilon toxin mutant Y30A-Y196A raised in rabbits at 1:500 dilution as described in Bokori-Brown M, et al. Clostridium perfringens epsilon toxin mutant Y30A-Y196A as a recombinant vaccine candidate against enterotoxemia. Vaccine 32, 2682-2687 (2014), followed by IRDye® 800CW goat (polyclonal) anti-rabbit IgG (H+L) secondary antibody according to the manufacturer's recommendations (LI-COR Biosciences). The conjugate has been specifically tested and qualified for Western blot.<br>For loading control we used the mouse monoclonal Na+/K+ - ATPase α1 (C464.6): sc-21712 primary antibody (Santa Cruz Biotechnology) at 1:200 dilution according to the manufacturer's recommendations (https://datasheets.scbt.com/sc-21712.pdf) followed by IRDye® 680RD goat (polyclonal) anti-mouse IgG (H+L) secondary antibody according to the manufacturer's recommendations (LI-COR Biosciences). The conjugate has been specifically tested and qualified for Western blot. For accurate quantitation of oligomer yields we performed Western blot of a two-fold dilution series of cell lysates (ranging from 42-0.65 microgram). This verified that we are working within the linear range of detection and signal intensity is proportional to sample loading. Source data are provided as a Source Data file.

Eukaryotic cell lines

Policy information about cell lines

Cell line source(s)	Super Dome cell line (ATCC® CRL-2286™) was sourced from the American Type Culture Collection (ATCC), PO Box 1549, Manassas, VA 20108 USA
Authentication	The authenticated Super Dome cell line (ATCC® CRL-2286™) was sourced from ATCC, a recognisable cell bank.
Mycoplasma contamination	Super Dome cell line (ATCC® CRL-2286™) tested negative for mycoplasma contamination performed by Eurofins.
Commonly misidentified lines (See ICLAC register)	This study did not use any commonly misidentified cell lines.
