# Supplementary material for: The pore structure of Clostridium perfringens epsilon toxin
Source: Nat Commun. 2019 Jun 14;10:2641. doi: 10.1038/s41467-019-10645-8 (PMC6572795; doi:10.1038/s41467-019-10645-8)
Supplement: Supplementary file 4 — Description of Additional Supplementary Files [file 41467_2019_10645_MOESM4_ESM.docx]

**Title: Supplementary Movie 1**

**Description:** Animation of the conformational changes during the transition from monomeric Etx to the membrane-inserted form: Etx monomers are proteolytically activated by removal of the C-terminal peptide (CTP, shown is stick representation). Once the CTP is removed, Etx can oligomerise into a pre-pore and transition into the pore form by the large conformational changes that occur in the cap domain (yellow) and the β-hairpin domain (blue), while the receptor binding domain (green) remains largely unchanged. Finally, the cryo-EM density is shown (transparent grey) along with the atomic model.
